# Increased Expression of IL-37 in Patients with Graves' Disease and Its Contribution to Suppression of Proinflammatory Cytokines Production in Peripheral Blood Mononuclear Cells

**DOI:** 10.1371/journal.pone.0107183

**Published:** 2014-09-16

**Authors:** Yanqun Li, Zi Wang, Ting Yu, Bingni Chen, Jinshun Zhang, Kunzhao Huang, Zhong Huang

**Affiliations:** 1 Biological therapy institute, Shenzhen University School of Medicine, Shenzhen, China; 2 Department of Pathogen biology and immunology, Shenzhen University School of Medicine, Shenzhen, China; 3 Shenzhen City Shenzhen University Immunodiagnostic Technology Platforms, Shenzhen, China; University of Michigan Medical School, United States of America

## Abstract

**Background:**

Intreleukin-37 (IL-37), a member of IL-1 family, is primarily an anti-inflammatory cytokine, which reduces systemic and local inflammation. However, the expression and role of IL-37 in Graves' disease (GD) remains unknown. This study aims to measure the levels of serum and peripheral blood mononuclear cells (PBMCs) IL-37 in patients with Graves' disease and to examine its association with disease activity. Furthermore, we investigate the effect of IL-37 on proinflammatory cytokines involved in the pathogenesis of GD.

**Methods:**

The expressions of IL-37, TNF-α, IL-6, and IL-17 mRNA in peripheral blood mononuclear cells (PBMCs) of 40 patients with Graves' disease were determined by real-time reverse transcription-polymerase chain reaction (RT-PCR), and the levels of IL-37, TNF-α, IL-6, and IL-17 in serum were detected by enzyme-linked immunoassay (ELISA). The correlation of serum IL-37 levels with cytokines and disease activity in Graves' disease patients were investigated. The expressions of cytokines TNF-α, IL-6, and IL-17 in PBMCs under recombinant IL-37 stimulation were determined by RT-PCR and ELISA respectively.

**Results:**

The levels of IL-37, TNF-α, IL-6, and IL-17 in PBMCs and serum were significantly increased in patients with GD compared with healthy controls (HC). Serum IL-37 were closely correlated with TNF-α, IL-6, IL-17, thyrotropin (TSH), free thyroxine (FT4),free triiodothyronine (FT3) and thyrotropin receptor antibody (TRAB). GD patients with active disease showed higher IL-37 mRNA and serum protein levels compared with those with inactive disease as well as HC. Moreover, IL-37 suppressed the production of IL-6, IL-17 and TNF-α in PBMCs of patients with GD.

**Conclusions:**

Increased level of IL-37 in patients with GD are associated with TNF-α, IL-6, IL-17 and disease activity, and it plays a protective role against inflammatory effect in GD by inhibiting the production of proinflammatory cytokines. Thus, IL-37 may provide a novel research target for the pathogenesis and therapy of GD.

## Introduction

Graves' disease (GD) is an organ-specific autoimmune disease characterized by the production of agonistic auto antibodies against the thyroid-stimulating hormone receptor (TSHR), which mimic the stimulatory effects of TSH, leading to hyperthyroidism and diffuse hyperplasia of the thyroid gland [1]–[3]. GD is the most common cause of Hyperthyroidism [4]. Although the pathogenesis of the disease remains elusive, evidences indicated that destruction of the balance of pro- and anti- inflammatory cytokines result in thyroid lymphocytic infiltration and B cell activation that produce autoimmune antibodies against thyroid antigens, which in turn played an important role in the pathogenesis of GD [1]. Abundant studies have illuminated that pro-inflammatory cytokines including IL-2, IL-8, IL-6 [5]–[11], TNF-α [12]–[14] and IL-17 [15]–[16] were increased in the serum of patients with GD. Of note, intrathyroidal lymphocytes secrete pro-inflammatory cytokines, such as IL-1 and TNF-α, which involve in the intrathyroidal autoimmune response in GD [1]. The previous study demonstrated that thyroid cells produce pro-inflammatory cytokines, such as TNF-α, IL-1 and IFN-γ, and they further exacerbate inflammatory reaction in GD [1]. It has been shown that antithyroid drugs reduce the production of thyroidal pro-inflammatory cytokines contributing to remit the inflammation of GD [1]. It also have been found that a high level of IL-6 in patients with untreated GD and returned to normal after treatment [9]. Recent research shown that the percentages of Th17 and the levels of plasma IL-17 in GD patients were correlated positively with the levels of serum TRAb in these patients, suggesting that IL-17 may contribute to the pathogenesis of GD in Chinese [15].Serum IL-6, TNF-α and IL-17 levels correlated strongly with thyroid endocrine status and disease activity, indicating that they play pivotal roles in the pathogenesis of GD [17]–[19].

IL-37, a new member of IL-1 family, originally named as IL-1 family member 7 (IL-1F7). There are five different splice variants of IL-37 termed IL-37a-e [20]–[24]. Isoform IL-37b is the largest isoform and has the most complete set of exons. It has significant sequence similarity with IL-18 and is the most studied [25].

Recently, IL-37 has been proved to be a natural suppressor of innate immunity and inflammatory responses. The published study suggested that IL-37 is highly expressed in inflammatory tissues, which inhibits the excessive inflammatory response [20]. It has been demonstrated that IL-37 can be induced by several toll-like receptor (TLR) ligands and pro-inflammatory cytokines such as IL-1β, TNF-α, IFN-γ in PBMCs [20], [26]. However, over-expressed human IL-37 inhibited the TLR-induced pro-inflammatory cytokines in mouse macrophage RAW cell line, human monocytic cell line THP-1 and epithelial cell line A549 [20], [27]. Researchers showed that IL-37 transgenic mice (IL-37tg) can markedly reduce clinical manifestations of DSS colitis, ischemia–reperfusion injury, obesity-induced inflammation, LPS-induced shock and psoriasis, and ameliorate their inflammatory cytokine productions [28]–[31]. On the other hand, Imaeda at el has expounded and proved that IL-37 is detected in the inflamed mucosa of IBD patients but not in the health controls [32], Zhong-Yuan Wan at el found that down-regulation of IL-37 expressions appeared to result in over-expression of pro-inflammatory cytokines, such as IL-1β and IL-16 in degenerative intervertebral disc, and they believed that IL-37 may have function to delay the progression of intervertebral disc degeneration [33]. Our previous study also showed that IL-37 can inhibit PBMCs of systemic lupus erythematosus (SLE) patient to produce pro-inflammatory cytokines, and its expressions are positively associated with SLE renal disease activity [34].

However, the level of IL-37 in GD patients and how the expression of IL-37 related to GD have not been explored. In this study, we compared expression of IL-37 mRNA in PBMCs and serum IL-37 protein levels in Chinese GD patients with healthy controls. Moreover, we analyzed the association of serum IL-37 with disease activity and inflammatory cytokines in these GD patients, investigated the effects of IL-37 on the expression of inflammatory cytokine TNF-α, IL-6 and IL-17 in GD patient's PBMCs.

## Materials and Methods

### Ethics Statement

This study was approved by the Review Board of Peking University Shenzhen Hospital in Shenzhen, People's Republic of China. Written informed consent was obtained from all participants.

### Patients and Healthy Individuals

Forty patients were recruited randomly from the Peking University Shenzhen Hospital in Shenzhen, People's Republic of China. Individual patients with GD were diagnosed according to the clinical evidences of hyperthyroidism that include the presence of typical symptoms (such as weight loss despite increased appetite,fatigue, heat intolerance, increased sweating, tremors and so on.); and the laboratory diagnosis that include decreasing serum TSH, increasing FT3, FT4, TRAb; (TSH<0.34 uIU/L, normal range: 0.34–5.6 pmol/L; FT3>6.30 pmol/L, normal range:3.3–6.3 pmol/L; FT4>24.5 pmol/L, normal range: 10.3–24.5 pmol/L; TRAb>30 IU/mL, normal range: 0.11–30 IU/mL.).

According to medical history recall, all GD patients have been treated with Propylthiouracil or Methimazole for 2 months at least. As a result, some of their TRAb levels decrease to normal range, but their symptoms did not remit and the TSH still below the lower limit of normal range. Thirty gender- and age-matched healthy volunteers without a family history of GD were recruited from the outpatient service of the same hospital and served as healthy controls (HC). Individual participants with current or a history of other autoimmune diseases or other chronic inflammatory diseases were excluded from the study. Demographic and clinical information of the study subjects are presented in Table 1.

**Table 1:** The demographic and clinical characteristics of the participants.

Characteristics	GD patients	Active GD	Inactive GD	Healthy controls
Number	40	14	26	30
Age(years)	37.73±12.32	36.86±12.35	38.19±12.52	36.80±10.49
Sex(female/male)	30/10	12/2	18/8	20/10
Disease duration(years)	2.81±1.23	2.61±1.11	2.91±1.29	-
Diffuse goiter n (%)	5(12.5)	5(35.71)	0(0)	-
Ophthalmopathy n (%)	3(7.5)	2(14.29)	1(3.85)	-
TSH (mIU/Ml)	0.03±0.02	0.02±0.02	0.03±0.02	0.34–5.6
FT4 (pmol/L)	22.50±28.17	50.64±31.25	7.34±6.92	10.3–24.5
FT3 (pmol/L)	19.53±17.20	20.93±19.15	18.17±16.40	3.3–6.3
TRAB(IU/mL)	20.64±7.77	23.69±10.08	19.00±5.77	0.11–30
TgAb(IU/mL)	249.83±248.85	354.50±335.63	193.47±169.09	<30
TPO-AB(IU/mL)	392.32±282.56	538.53±293.61	316.29±249.76	<30
Treatment(mg)	Range:50∼150	Range:50∼150	Range:50∼150	-

TSH, thyrotropin; FT4, free thyroxin; FT3, free triiodothyronine; TRAB, thyrotropin receptor antibody; TPOAb, thyroid peroxidase antibody; TgAb, thyroglobulin antibody; Data shown are the case number or median (range). (mg,Propylthiouracil,Methimazole).

FT3, FT4, TSH and TPOAb were measured by automated chemiluminescent immunoassays (Unicel Dxi800; Beckman Coulter); TGAb and TRAb were measured by radioreceptor assays with commercial kits (Beckman Coulter, Brea).

### Blood sample

Blood samples were collected from peripheral veins. PBMCs were isolated by a Ficoll-Paque Plus (TBD science, China) density gradient centrifugation for cell culture or stored at −80°C until RNA extraction. Serums were frozen at −80°C until cytokines were detected.

### Recombinant human IL-37 protein

In our previous work, we have cloned, expressed and purified human IL-37 protein, and identified its functional activity [34].

### Cell culture condition

Culture medium, consisted of RPMI 1640(Hyclone, Thermo, USA) supplemented with 10% Fetal Calf Serum (Sijiqing, China), 100 IU/ml penicillin and 100 µg/ml streptomycin, respectively. Whole PBMCs were cultured in 24 well, flat-bottomed plates for 24 h. PBMCs were stimulated with or without human recombinant IL-37 at 100 ng/ml for 4 h, then, incubated further with LPS (1 µg/ml) for 6 h [34], total RNAs were extracted, and cytokine transcriptions were analyzed by RT-PCR. For cytokine protein analysis, PBMCs were stimulated with or without human recombinant IL-37 at 100 ng/ml for 24 h, and then incubated further with LPS (1 µg/ml) for 6 h [34], culture supernatants were harvested and frozen at −80°C for later cytokine analysis by ELISA.

### Real-time PCR

Total RNAs were extracted from PBMCs by using TRIzol (Invitrogen,USA). The quantity and purity of RNAs were determined by absorbance on Epoch Multi-Volume Spectrophotometer System (Biotek, USA) at 260 nm and 280 nm. Samples with ratios from 1.8 to 2.0 were accepted for next reverse transcription reaction. cDNAs were synthesized by using the iScript cDNA Synthesis Kit (Thermo, USA) according to manufacturer's instructions. Each real-time PCR was prepared in a 20 µL reaction mixture and conducted on a CFX96 Real-Time System (Bio-rad, USA). The PCR primer (Generay, China) sequences are summarized in Table 2. PCR products were verified by melting curve analysis. Relative mRNA levels of target genes were calculated with normalization to β-actin values using the 2^−ΔΔct^ method.

**Table 2:** List of the sequence of human gene primers.

Gene name	Forward (5′ to 3′)	Reverse (5′ to 3′)
IL-6	AGCCACTCACCTCTTCAGAAC	ACATGTCTCCTTTCTCAGGGC
IL-17	CCCGGACTGTGATGGTCAAC	GCACTTTGCCTCCCAGATCA
TNF-α	ACCTCTCTCTAATCAGCCCTCT	GGGTTTGCTACAACATGGGCTA
IL-37	AGTGCTGCTTAGAAGACCCGG	AGAGTCCAGGACCAGTACTTTGTGA
actin-β	CCTGACTGACTACCTCATGAAG	CGTAGCACAGCTTCTCCTTA

### ELISA

Serum IL-37, TNF-α, IL-6 and IL-17 levels and cell culture supernatant IL-37, TNF-α, IL-6 and IL-17 levels were determined by ELISA following the manufacturer's instructions respectively. Human IL-37 ELISA reagent Kits were purchased from Cusabio (Wuhan, China); Human TNF-α, IL-6 and IL-17 ELISA reagent Kits were purchased from eBioscience (San Diego, CA, USA).

### Statistical analysis

Data were expressed as mean (± SEM) or median (range) and analyzed by Graphpad Prism V.5.00 software (GraphPad Software, San Diego CA, USA). Differences between control and treated groups were compared by Mann-Whitney-U-test. Spearman correlation test was used to assess the association between serum IL-37 levels and different variables. P values under 0.05 were considered statistically significant.

## Result

### Increased serum levels of IL-37 and inflammatory cytokines in GD patients

**Figure 1:**
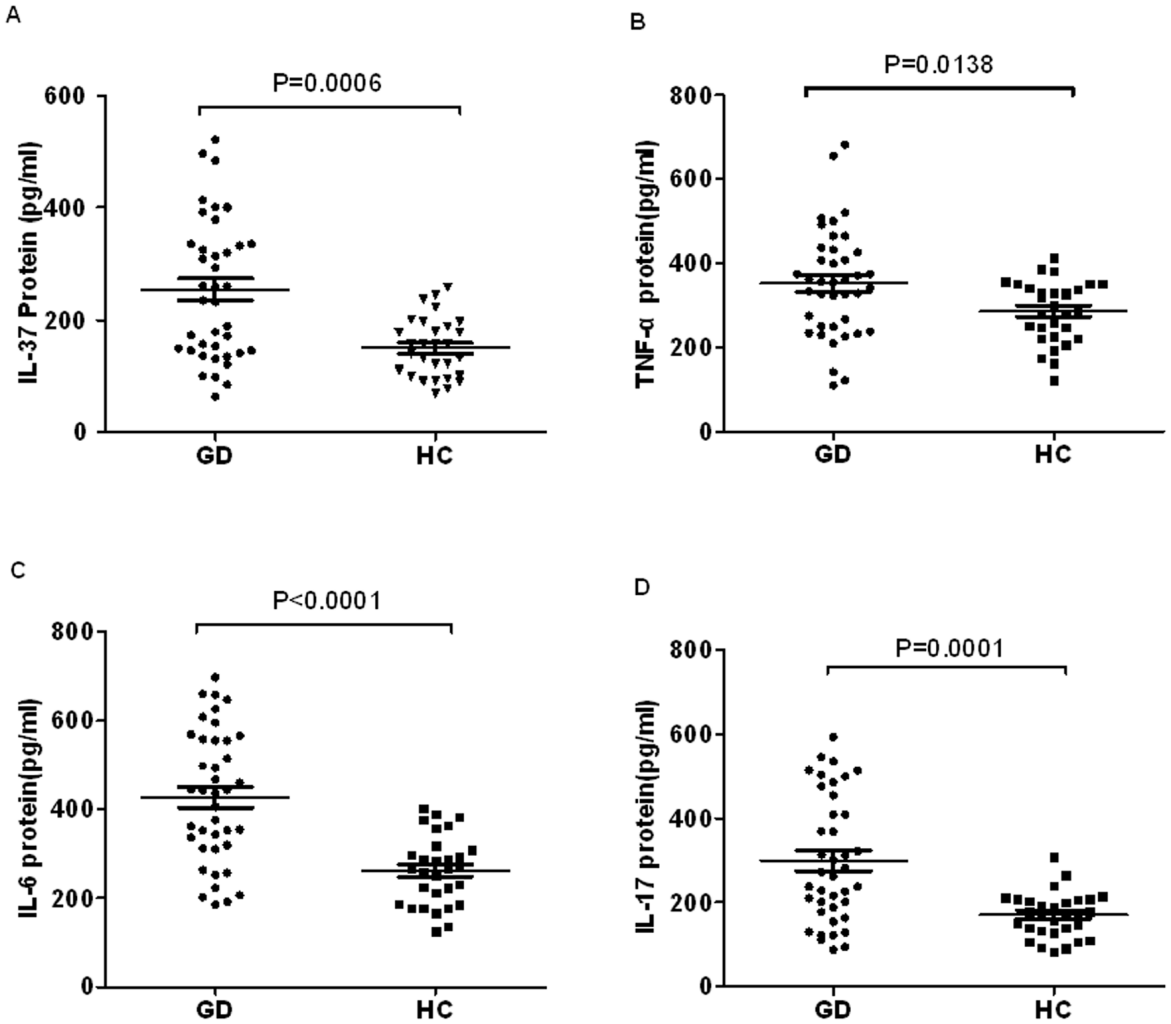
Comparison the protein levels of IL-37 and inflammatory cytokines between GD patients and HCs. (A) Serum IL-37 protein levels in GD patients (n = 40) and healthy controls (HC, n = 30) were determined by ELISA. (B) Serum cytokine TNF-a, IL-6 and IL-17 concentrations in GD patients (n = 40) and HCs (n = 30) were analyzed with ELISA. Each symbol represents an individual GD patient and HC. Horizontal lines indicate median values. Mann-Whitney U-test and associated p values are indicated.

The demographic and clinical features of GD patients and controls are reported in Table 1. Total 40 GD patients and 30 HC with age and sex matched were involved in this study. To compare the protein level of IL-37 between the patients with GD and HC, ELISA were used to measure serum IL-37. The results showed that serum IL-37 levels in GD patients were significantly higher than those from HC (Fig. 1A, p = 0.0006). To investigate the protein levels of major pro-inflammatory cytokines involved in GD pathogenesis, the serum levels of TNF-α, IL-6 and IL-17 were measured by ELISA in GD patients and HC, respectively; Pro-inflammatory cytokine TNF-α (Fig. 1B, P = 0.0138), IL-6 (Fig. 1C, P<0.0001) and IL-17 (Fig. 1D, P = 0.0001) serum levels in GD patients were significantly higher than those from HC. Moreover, spearman correlation analysis revealed that serum IL-37 levels of GD patients were positively correlated with the levels of pro-inflammatory cytokine TNF-α, IL-6 and IL-17(Table 3). These results showed that IL-37 protein levels were elevated in GD patients and positively related to pro-inflammatory response of GD.

**Table 3:** Correlation between serum IL-37 levels and inflammatory cytokines as well as laboratory values.

Parameter	correlation coefficient (r)	P-value
TNF-α	0.4386	0.0046
IL-6	0.3866	0.0137
IL-17	0.5081	0.0008
TSH (mIU/mL)	−0.3945	0.0118
FT4 (pmol/L)	0.4413	0.0044
FT3 (pmol/L)	0.3318	0.0365
TRAB (IU/mL)	0.3645	0.0207
TgAb (IU/mL)	0.1775	0.2732
TPO-AB (IU/mL)	0.2991	0.0608

### Up-regulated expressions of IL-37mRNA and inflammatory cytokines in PBMCs of GD patients

**Figure 2:**
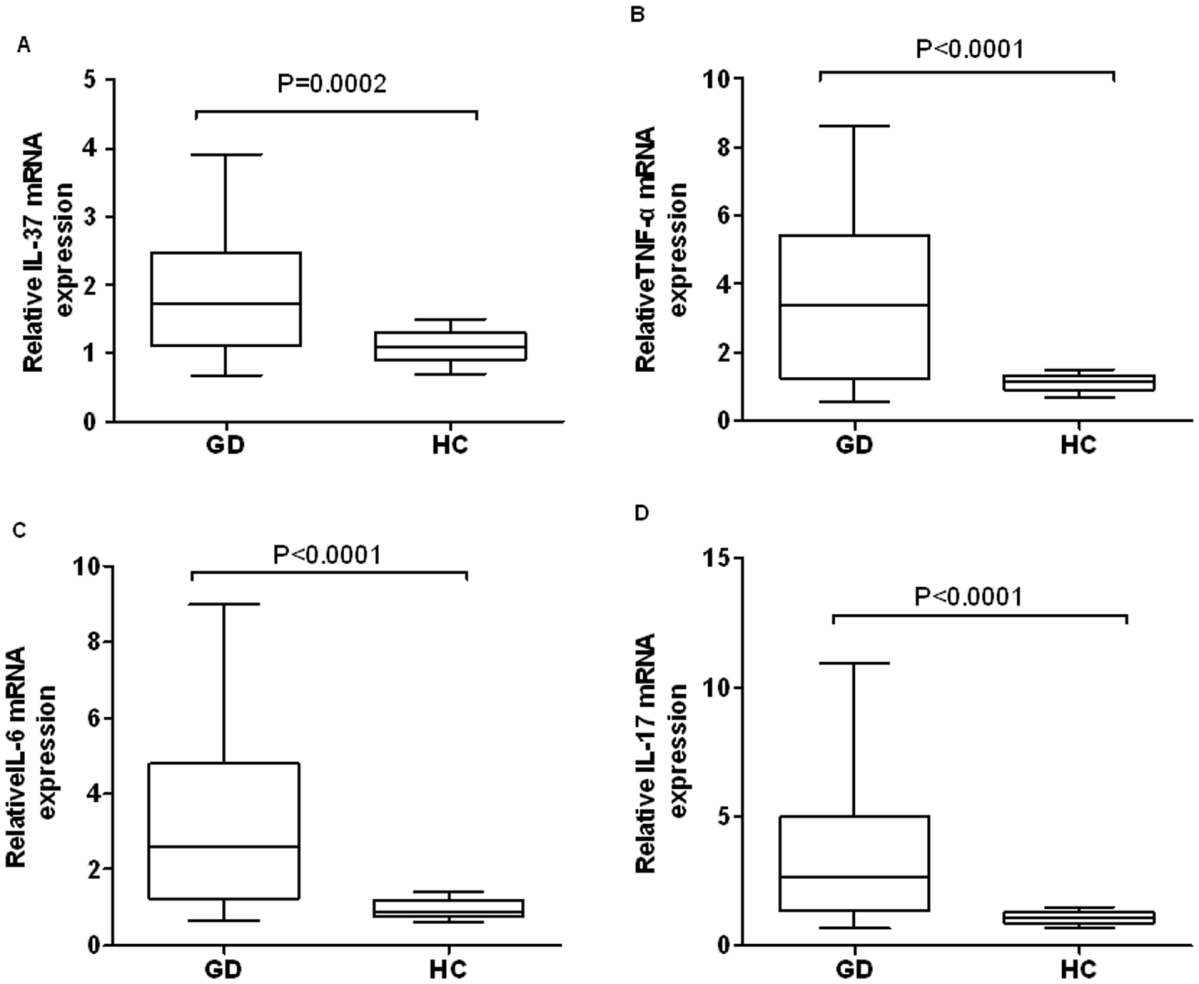
Comparison of mRNA levels of IL-37 and cytokines between GD patients and HCs. (A) Expressions of IL-37 mRNA in PBMC from GD patients (n = 40) and HCs (n = 30) were determined by RT-PCR. (B) mRNA levels of cytokines TNF-a,IL-6 and IL-17 in GD patients (n = 40) and HCs (n = 30) were analyzed with RT-PCR. Results were depicted as box plots, with median (horizontal line within each box) and 10th, 25th, 75th, and 90th percentiles (bottom bar, bottom of box, top of box, and top bar, respectively).

The expressions of IL-37 and inflammatory cytokines in PBMCs were investigated in GD patients and HCs. As shown in Fig. 2A, compared with HC, the expression of IL-37 mRNA were significantly higher in PBMCs of patients with GD (P = 0.0002). Consistently, the expression of pro-inflammatory cytokines TNF-α (Fig. 2B, P<0.0001), IL-6 (Fig. 2C, P<0.0001) and IL-17 (Fig. 2D, P<0.0001) were also notably higher in GD patients than in HCs. These results indicate that the expression of IL-37 in PBMCs was positively correlated with the expression of pro-inflammatory cytokines TNF-α, IL-6 and IL-17 in GD patients.

### IL-37 mRNA and serum protein levels were higher in GD patients with active disease compared with those with inactive disease

**Figure 3:**
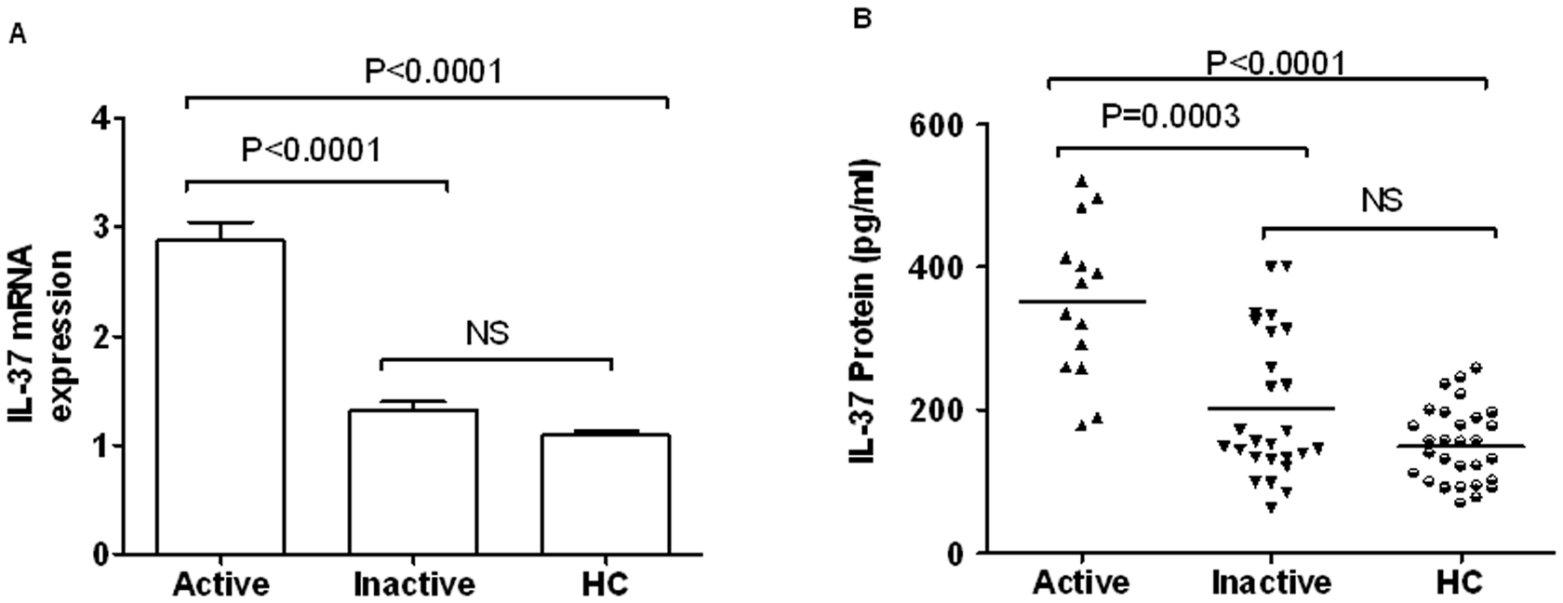
mRNA and protein levels of IL-37 in active and inactive GD as well as HC. According to the levels of TSH and FT4, the study GD patients were divided into disease activity (n = 14) and inactive (n = 26) groups. (A) Serum IL-37 protein levels were detected by ELISA in active and inactive GD patients as well as HCs (n = 30). Bars show the expression levels of IL-37 in different groups (mean ± SEM). (B) Levels of IL-37 mRNA in PBMCs from active (n = 14) and inactive (n = 26) GD patients as well as HCs (n = 30) were measured by RT-PCR. Each symbol represents an individual inactive, active patient or healthy control. Horizontal lines indicate median values. Mann-Whitney U-test and associated P values are indicated.

To further evaluate whether the expression of IL-37 was related to disease activity in GD patients. We divided GD patients into inactive GD (low TSH with normal FT4 and FT3 or part of them with high FT3) and active GD (low TSH with high FT4) (Table 1) according to the severity of hyperthyroidism [1], [36], [37]. As seen in Fig. 3, active group of GD conferred significantly higher levels of IL-37 mRNA and protein than inactive group (P<0.0001; P = 0.0003) in PBMCs and serum respectively. Furthermore, GD patients with active disease displayed higher IL-37 mRNA of PBMCs and serum IL-37 levels than HC (P<0.0001; P<0.0001, respectively). However, we did not observe the differences of IL-37mRNA and protein levels between patients with inactive disease and HC. These results show that the expressions of IL-37 are corresponded to the disease activity of GD. From which, we inferred that the inflammatory reaction of GD is associated with the up regulation of IL-37 in PBMCs.

### Association between IL-37 levels and laboratory values

The production of agonistic autoantibodies that over-stimulates the production of thyroid hormones is a pathogenic hallmark of GD. We further analyzed the relevance between serum IL-37 levels and the concentrations of TSH, FT3, FT4, TRAb, TgAb and TPO-Ab in GD patients. As show in Table 3, we found that serum IL-37 were positively related to FT3, FT4 and TRAb, but there was a negative correlation between serum IL-37 and TSH levels. No significant correlations were found between serum IL-37 and TgAb or TPO-Ab. It indicates that the levels of IL-37 correlate to the laboratory values of active GD.

### IL-37 suppressed the production of pro-inflammatory cytokines in PBMCs of GD patients

**Figure 4:**
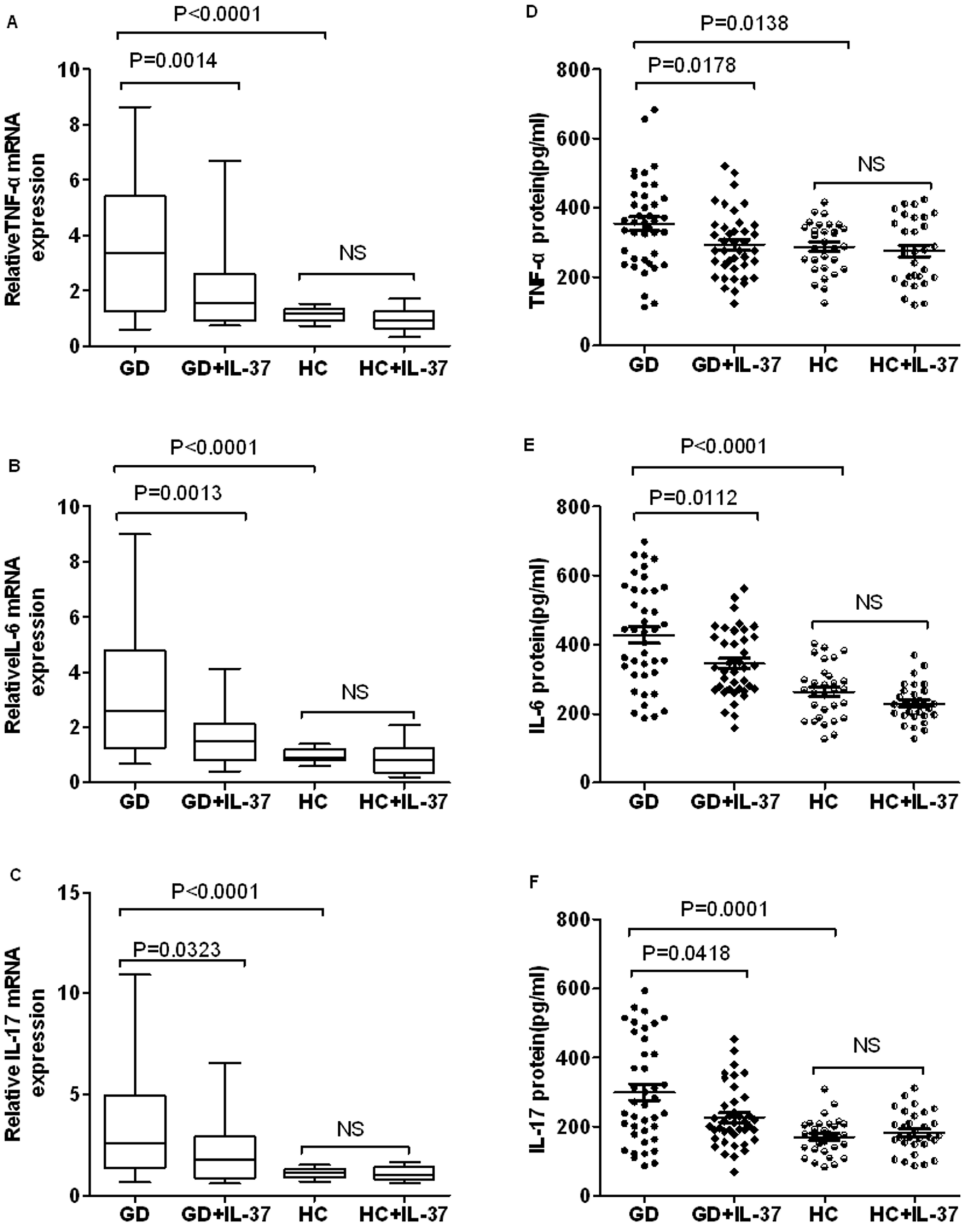
IL-37 inhibits the expression of inflammatory cytokines in PBMCs of patients with GD. PBMCs from GD patients (n = 40) and HCs (n = 30) were stimulated with recombinant IL-37 (100 ng/ml) for 4 h, total RNAs were extracted and analyzed for TNF-α(A), IL-6(B) and IL-17(C) mRNAs by RT-PCR. Results were depicted as box plots, with median (horizontal line within each box) and 10th, 25th, 75th, and 90th percentiles (bottom bar, bottom of box, top of box, and top bar, respectively). The PBMCs of GD patients and HCs from above the same groups were stimulated with recombinant IL-37 (100 ng/ml) for 24 h and then further stimulated with LPS(1 ng/mL) for 6 h, supernatants were collected and examined for TNF-α (D), IL-6 (E) and IL-17 (F) levels by ELISA. Each symbol represents an individual patient or healthy control. Horizontal lines indicate median values. Actual P values are shown in the graph, NS, no significant.

**Figure 5:**
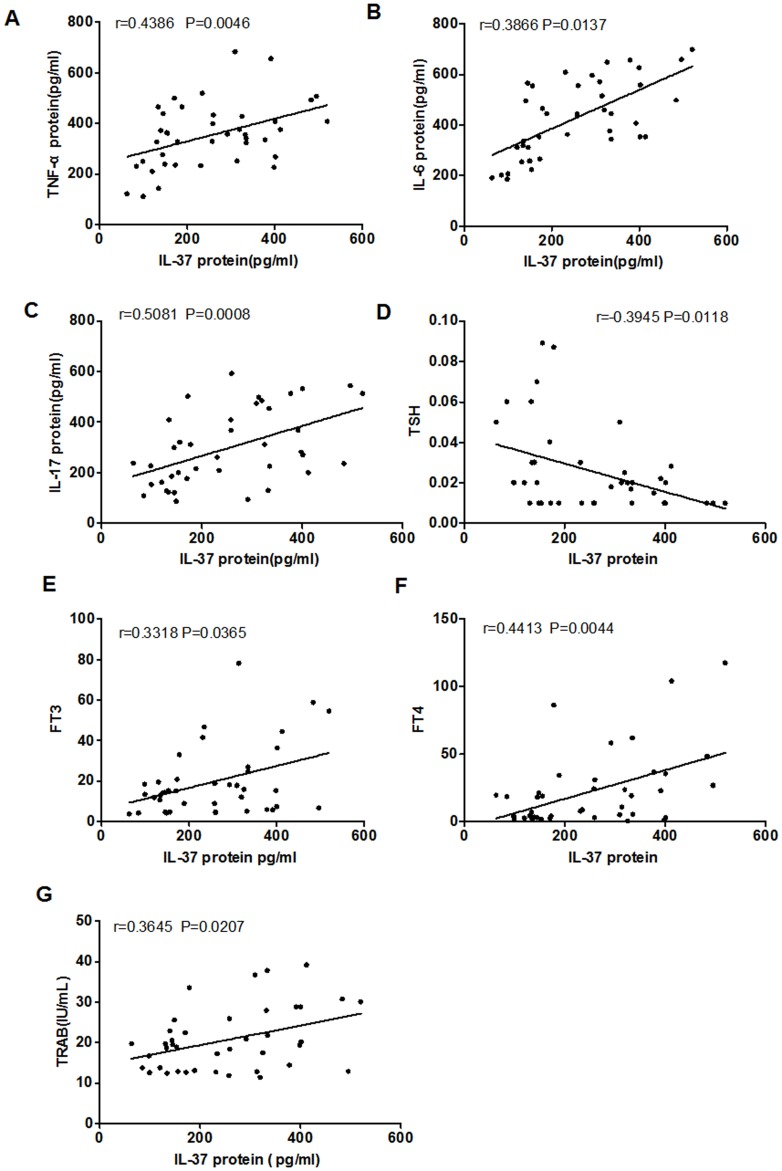
Correlation of serum IL-37 levels with pro-inflammatory cytokines as well as laboratory values. Each symbol represents an individual GD patient. (A–C) Serum IL-37 levels were positively correlated with pro-inflammatory cytokine TNF-a, IL-6, IL-17. (D) Negative relationship was observed between serum IL-37 levels and TSH. (E-G) Positively relationship was observed between serum IL-37 levels and FT3, FT4, TRAB. The correlations were evaluated with Spearman's non-parametric test.

It has been testified that IL-37 inhibited the excessive inflammatory response in autoimmune diseases. To investigate whether the elevated IL-37 was responsible for the down-regulations of pro-inflammatory cytokines in PBMCs in GD patients, PBMCs from GD patients and HC were cultured and either treated or untreated with purified recombinant human IL-37 protein at concentrations 100 ng/ml for 4 h and 24 h respectively, and then incubated further with LPS (1 µg/ml) for 6 h. The cells and cultural supernatants were harvested for RT-PCR and ELISA analysis, separately. Compare to HC, treatment with recombinant IL-37 significantly suppressed the expressions of TNF-α (Fig. 4A, P = 0.0014), IL-6 (Fig. 4B, P = 0.0013) and IL-17 (Fig. 4C, P = 0.0323) in PBMCs of GD patients. Moreover, Treatment with recombinant IL-37 also markedly reduced the secretion of pro-inflammatory cytokines TNF-α (Fig. 4D, P = 0.0178), IL-6 (Fig. 4E, P = 0.0112) and IL-17 (Fig. 4F, P = 0.0418) in PBMCs of GD patient, but the expression of these cytokines in PBMCs of HC were unaffected by treatment with recombinant IL-37. Combined with the correlation between IL-37 and inflammatory cytokine IL-6, IL-17 and TNF-a (Fig. 5A, 5B, 5C), These results suggested that IL-37 involve in the inflammatory mechanism of GD and suppress excessive inflammation by inhibiting the expression of pro-inflammatory cytokines.

## Discussion

A growing body of evidence indicates that IL-37 inhibits inflammation reactions in autoimmune diseases, which are commonly associated with disease activities [20], [37]. Our previous evidence indicated that up-regulation of IL-37 mediates a feedback mechanism to suppress pro-inflammatory cytokine productions in patients with SLE [34]. However, the expression and role of IL-37 in GD remain to be elucidated. In this study, we found that IL-37 protein in serum and its mRNA expression levels in PBMCs were significantly higher in GD patients than in HCs (Fig.1A and Fig. 2A). Thus, our results indicated IL-37 involved in the regulation of GD inflammatory reaction.

Published data has been demonstrated that IL-37 can alleviate the symptoms of DSS colitis and inflammatory process in psoriasis by inhibiting the expression of inflammatory cytokines in these diseases [28], [31] and reduce liver inflammatory injury via effects on hepatocytes and non-parenchymal cells [29]. It has been demonstrated that IL-37 has the properties of reducing obesity-induced adipose tissue inflammation and improve insulin sensitivity [30].

To study the correlation between IL-37 and major pro-inflammatory cytokines of GD, the quantities of TNF-α, IL-6, IL-17 were measured by ELISA and RT-PCR. Our results confirmed that the expressions of these pro-inflammatory cytokines were increased in GD patients (Fig. 1B, C, D and Fig. 2B, C, D). More importantly, our experiments showed that the levels of serum IL-37 and expressions of IL-37 in PBMCs were positively related to the levels of TNF-α, IL-6, IL-17 in GD patients.

A characteristic feature of GD is the lymphocytic infiltration and the production of large numbers of pro-inflammatory and anti-inflammatory cytokines by thyroid cells [1]. IL-6 can be expressed by number of cells, including thyroid cells. Experiments have shown that IL-6 concentrations in plasma are high in patients with sub-acute thyroiditis [38]. Siddiqui and colleagues found a high level of IL-6 in patients with untreated GD and returned to normal after treatment [9]. Serum TNF-a levels are elevated in patients with GD [39]. TNF-a has been suggested as a possible mediator of increased expression of MHC class I molecules on thyroid epithelial cells in GD. Combined with IFN-γ, TNF-a enhances the induction of MHC class II antigens on the thyroid cells *in vitro* [40], [41]. The polymorphisms in the TNF-a gene promoter have been shown to associate with the increased incidence of GD [42], [43]. Thus, TNF-a signaling is important for the pathogenic development of GD. Di Peng et al found significantly higher percentages of peripheral blood Th17 and higher levels of plasma IL-17 in GD patients. The percentages of Th17 and the levels of plasma IL-17 were correlated positively with the levels of serum TRAb in these patients, suggesting that IL-17 may contribute to the pathogenesis of GD in Chinese [15]. Taken together, the published data have shown that the pro-inflammatory cytokine TNF-α, IL-6 and IL-17 play a pivotal role in the inflammatory reaction and development of GD.

Recent studies have shown that pro-inflammatory cytokine TNF-α, IL-1β, IL-18, IL-12 induce the expression of IL-37 in peripheral blood mononuclear cells (PBMCs) *in vitro* [25]. *In vivo*, it has been shown that the expressions of IL-37 were up-regulated by inflammatory activity and pro-inflammatory cytokines in autoimmune diseases, such as rheumatoid arthritis and SLE, especially in active stage of autoimmune disease [25], [34]. In our present study, the expressions of pro-inflammatory cytokines TNF-α, IL-6 and IL-17 were significantly higher in PBMCs of GD patients than in PBMCs from HCs (Fig. 1B, C, D and 2B, C. D). More importantly, our experiments showed that IL-37 suppress the production of pro-inflammatory cytokines IL-6, TNF-α and IL-17 in PBMCs of GD patients, but not in HCs (Fig.4).

Considering the pro-inflammatory cytokines in serum have been shown to involved in the pathogenesis of GD [1], [17]–[19], the pro-inflammatory cytokines have been demonstrated to stimulate the expression of IL-37 [25], protein and mRNA levels of IL-37 were positively correlated with pro-inflammatory cytokine TNF-α, IL-6 and IL-17 in GD patient serum and PBMCs (Fig. 1 and 2), and these pro-inflammatory cytokines were down regulated by IL-37 (Fig. 4). Thus, it is reasonable to speculate the pro-inflammatory cytokines in GD patients may involve in stimulating the expression of IL-37 in PBMCs, and IL-37 may mediate a negative feedback mechanism to suppress excessive pro-inflammatory cytokines in GD inflammation.

Our previous study showed the activity of SLE correlate with the expression levels of IL-37 [34].To investigate whether up-regulated serum IL-37 levels associated with GD disease activity, ELISA and RT-PCR were used to analyze the levels of IL-37 in active, inactive GD patients as well as HCs. The experiments indicated that the levels of IL-37 in serum and PBMCs both positively relate to the activity of GD (Fig. 3). Furthermore, spearman correlation test was used to assess correlation between IL-37 and disease activity in GD, and our data showed that the expression of IL-37 is positively correlated with the activity of GD patients (Table 3). These results imply that the inflammatory reaction of GD might stimulate the expression of IL-37 in PBMCs.

According to the level of TSH and FT4, the GD is divided into inactive GD (low TSH with normal FT4) and active GD (low TSH with high FT4,). Our experiments showed that serum IL-37 levels were positively correlated with FT3 and FT4. However, there was a negative correlation between serum IL-37 and TSH levels. Pro-inflammatory cytokines such as TNF-a and IL-6 have been shown to increase the concentration of T3 and suppress TSH release [44], [45]. Recently, Th17 has been described both in mice and humans to favor the production of the pathogenic antibody directed against the TSH receptor, which constitutively activate the thyroid gland to produce T3 and T4 [46]. In our present study, TNF-α, IL-6 and IL-17 were increased in GD patients and positively correlated with the serum IL-37 levels, especially in active stage of GD patients. Giving the results of the IL-37 inhibits the expression of TNF-α, IL-6 and IL-17 in GD, we infer that increasing IL-37 plays direct and indirect roles in inhibiting the production of T3 and T4 and reduces the symptoms of GD.

In summary, our data have presented new evidence that the expression of IL-37 is correlated with the disease activity of GD, and contribute to suppress inflammation cytokines TNF-α, IL-6, and IL-17 during GD pathogenesis, and may play an inhibition role in the inflammatory reaction by reducing the productions of pro-inflammatory cytokine expressions. However, due to the limitation of human study, more studies should be performed to examine the detail molecular mechanisms in order to fully elucidate the regulatory network of IL-37 in GD.
